# Ovomemolins: Egg‐derived peptides that improved cognitive decline after oral administration in mice

**DOI:** 10.1096/fba.2023-00149

**Published:** 2024-06-10

**Authors:** Takanobu Nakajima, Maiko Shobako, Kentaro Kaneko, Atsushi Kurabayashi, Masaru Sato, Kousaku Ohinata

**Affiliations:** ^1^ Division of Food Science and Biotechnology, Graduate School of Agriculture Kyoto University Kyoto Japan; ^2^ Department of Applied Genomics Kazusa DNA Research Institute Kisarazu Japan

**Keywords:** bioactive peptide, cognitive decline, egg white, high‐fat diet, orally active, α7nAChR

## Abstract

Eggs not only contain all the molecules necessary to nurture new life but are also rich in nutrients such as high‐quality protein. For example, epidemiologic studies have shown that egg intake is positively correlated with cognitive function. Thus, we specifically examined the effect of ovalbumin, a major protein present in egg whites, on cognitive function. First, we found that an orally administered enzymatic digest of ovalbumin improves cognitive function in mice fed a high‐fat diet. Then, we narrowed down candidate peptides based on the prediction of peptide production according to enzyme‐substrate specificity and comprehensive peptide analysis of the digest. We found that three peptides, namely ILPEY, LYRGGLEP, and ILELP, improve cognitive function after oral administration. We also showed that ILPEY, LYRGGLEP, and ILELP were present in the digest and named them ovomemolins A (OMA), B, and C, respectively. Notably, ovomemolins are the first peptides derived from egg whites that have been shown to improve cognitive function. The cognitive improvement induced by OMA, the most abundant of the peptides in the digest, was inhibited by methyllycaconitine, an antagonist of α7nAChR, which is known to be related to memory. These results suggest that OMA improves cognitive function through the acetylcholine system. After OMA administration, brain‐derived neurotrophic factor (BDNF) mRNA expression and the number of 5‐bromo‐2′‐deoxyuridine‐positive cells suggested that OMA increases hippocampal *BDNF* expression and neurogenesis.

## Graphical abstract

We found that an enzymatic digest of ovalbumin, the major egg protein, ameliorates cognitive decline when administered orally to mice. We searched for peptides that improve cognitive function from this enzyme digest and found three new orally active peptides, which we named ovomemolins A‐C. Ovomemolin A improved cognitive function depends on the acetylcholine system and increased *BDNF* expression and neurogenesis in the hippocampus. Ovomemolins are the first example of egg protein‐derived peptides that ameliorate cognitive decline.

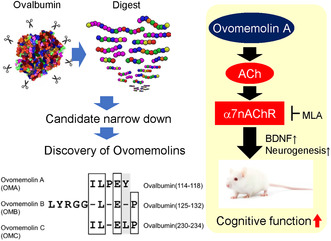

**Abbreviations**
ACh: acetylcholine
BDNF: brain‐derived neurotrophic factor
BrdU: 5‐bromo‐2′’‐deoxyuridine
CNTF: ciliary neurotrophic factor
EGF: epidermal growth factor
FGF‐2: fibroblast growth factor 2
GDNF: glial cell line‐derived neurotrophic factor
HFD: high‐fat diet
i.p.: intraperitoneal
ITT: insulin tolerance test
NGF: nerve growth factor
NT‐3: neurotrophin 3
OFT: open‐field test
OGTT: oral glucose tolerance test
OLT: object location test
OMA: ovomemolin A
ORT: object recognition test
p.o.: per os
α7nAChR: α7 nicotinic acetylcholine receptor

## 1 INTRODUCTION

The earliest record of the use of chicken eggs is found in a book written in ancient Egypt around 1500 BC, and a cookbook compiled around the fourth century contains recipes for soufflés and omelets. Eggs are still consumed throughout the world and are recognized as one of the main sources of protein, along with milk and meat. Containing all the molecules necessary to nurture new life, eggs are also a rich source of nutrients, such as high‐quality protein, and contribute to our health.

Moreover, the increasing prevalence of dementia imposes social and economic burdens and presents public health challenges. According to the World Health Organization, more than 55 million people currently have dementia worldwide, and the number is projected to nearly triple in 2050, compared with 2019.^1^ Although several drugs exist, novel ligands for the prevention and treatment of dementia must be developed.

One of the main risk factors that lead to dementia is impaired glucose tolerance. It has been reported that diabetic patients are 1.7 times more susceptible to dementia than the control group.^2^ Furthermore, we revealed that a high‐fat diet (HFD) induces glucose metabolism impairment and cognitive decline in mice.^3^, ^4^ Considering that milk intake is a protective factor, we found that tripeptide derived from α casein, a major component in bovine milk, ameliorated cognitive decline induced by HFD intake.^3^ The discovery of peptides derived from dairy protein sources that improve cognitive function may provide new measures of preventing cognitive decline, including mild cognitive impairment and dementia.^3^, ^5^ Notably, epidemiological studies have revealed a positive correlation between egg consumption and cognitive function.^6^ Thus, egg protein might be another suitable source of peptides. Herein, we focused on ovalbumin, a major protein found in egg whites, which have higher protein contents than egg yolks. To our best knowledge, no report has clearly demonstrated the association between ovalbumin and improvement of cognition; however, it cannot be rule out the possibility that ovalbumin may be a potential source of novel peptides that may mitigate cognitive decline.

In this study, we revealed that enzymatic digestion of ovalbumin improved cognitive function in mice. Thermolysin, which is a microorganism‐derived enzyme generally used for production on an industrial scale, was used herein. Next, we aimed to identify the cognitive‐improving peptides from the digest. Based on the prediction of peptide production according to the enzyme‐substrate specificity and comprehensive peptide analysis of the digest, we narrowed down the candidate peptides. Among them, we identified three peptides detected in the digest and referred to them as ovomemolins. These are the first peptides derived from egg protein reported to improve cognitive function. We also revealed the mechanism by which one of the peptides improves cognitive function.

## 2 MATERIALS AND METHODS

### 2.1 Reagents

Ovalbumin and thermolysin were purchased from Sigma‐Aldrich (St. Louis, MO, USA) and Nacalai Tesque (Kyoto, Japan), respectively. Ile‐Leu‐Pro‐Glu‐Tyr (ILPEY), Leu‐Tyr‐Arg‐Gly‐Gly‐Leu‐Pro‐Glu (LYRGGLPE), and Ile‐Leu‐Glu‐Leu‐Pro (ILELP), namely ovomemolins A (OMA), B (OMB), and C (OMC), respectively, were chemosynthesized using the F‐moc strategy and purified by reverse‐phase high‐performance liquid chromatography (HPLC). 5‐Bromo‐2′‐deoxyuridine (BrdU) was obtained from Sigma‐Aldrich (St. Louis, MO, USA). D‐Glucose was purchased from Wako Pure Chemical Industries (Osaka, Japan). Insulin (human) was purchased from Eli Lilly Japan K.K. (Hyogo, Japan).

### 2.2 Production of the ovalbumin digest

The ovalbumin digest was produced as described previously.^7^ After boiling the ovalbumin (20 mg/mL), thermolysin was added (E/S = 1/100), and the solution was incubated at 37°C for 5 h (pH 7.5). Then, the mixture was boiled to stop the reaction and centrifuged, and the supernatant was lyophilized and stored at −20°C.

### 2.3 Comprehensive analysis of the ovalbumin digest

The peptides in the ovalbumin digest were analyzed by nanoflow liquid chromatograph‐orbitrap high‐resolution mass spectrometry (nano‐LC–MS), as described previously^8^ but with slight modification. The peptides were purified using a GL‐Tip styrene‐divinylbenzene column (GL‐Science, Tokyo, Japan) and subsequently analyzed by nano‐LC–MS. The nano‐LC–MS system consisted of an Easy nLC II liquid chromatograph equipped with a PepMap RSLC C18 column, and a Q Exactive mass spectrometer (Thermo Fisher Scientific, Waltham, MS, USA). The peptides were separated by linear gradient elution of 0.1% formic acid in water and 0.1% formic acid in acetonitrile over 120 min. The nano‐LC–MS data were processed using Proteome Discoverer 2.2 (Thermo Fisher Scientific), with the ovalbumin protein sequence (Uniprot accession no. P01012) as a reference.

### 2.4 Peptide quantification

Each peptide concentration in the digest was quantified by ultra‐high‐performance liquid chromatography‐mass spectrometry (UPLC–MS) as described previously.^9^ The UPLC–MS setup consisted of an Acquity UPLC system (Waters, Milford, MA, USA) coupled to a Xevo quadrupole time‐of‐flight (QTOF) mass spectrometer (Waters) equipped with an electrospray ionization (ESI) source operating in positive‐ion mode, with a lock‐spray interface for accurate mass measurements. The concentration of ovomemolins in the ovalbumin digest was determined by comparison with the peak of the chemosynthesized standard peptides. In addition, the digest of 14‐amino acid residue model peptides was applied to the reverse‐phase HPLC column (C18‐AR‐II, Nacalai Tesque, Kyoto, Japan), and the peaks were collected. The peptide identification was performed by high‐resolution ESI‐QTOF, Xevo G2‐S qTof (Waters), and timsTOF (Bruker, Germany).

Pepsin and pancreatin digestion of OMs and LC‐MS analysis were performed in a similar manner to previous studies^10^ with slight modifications to simulate peptide bioavailability.

### 2.5 Animals

Herein, 10‐ to 11‐week‐old male ddY mice were purchased from SLC (Shizuoka, Japan). All mice were kept as previously described,^3^, ^4^ with slight modification. Rearing conditions of mice were established as follows: 23 ± 1°C on a 12‐h light–dark cycle with lights on at 7 a.m. Each mouse was acclimatized using a standard rodent diet, MF (Oriental Yeast, Osaka, Japan), for more than 6 days. After acclimatization, mice were divided into two experimental groups: the HFD group (Control group) and the HFD plus peptide administration group (HFD + peptide group). HFD intake was achieved by feeding each group with a 60 kcal% HFD (D12492, Research Diets Inc., New Brunswick, USA) beginning at the age of 12 weeks old. In the object recognition test (ORT) and object location test (OLT) schedules, both of the groups were fed HFDs for the initial 7 days, and the peptide was administered orally (p.o.: per os) at a dose of 3 mg/kg/day for an additional 5–7 days in the HFD + peptide group, whereas saline was administered in the HFD group.

For the BrdU incorporation and RNA preparation, both of the groups were fed the HFD for 10–11 days^3^, ^4^ and in the HFD + peptide group, the peptide was administered (3 mg/kg/day/p.o.) each day, but in the control group, saline was administered. During the administration period, the body weights were recorded each day for all mice. All experiments were approved by the Kyoto University Ethics Committee for Animal Research Use. All efforts were made to decrease the number of animals used and minimize their stress and pain.

### 2.6 Object recognition test (ORT) and object location test (OLT)

To evaluate cognitive function, the novel ORT and OLT were conducted on Days 6 and 7. The ORT was performed as previously described,^3^, ^4^, ^11^ with slight modification. Each mouse explored these objects similarly in the open field for 20 s, and the time spent exploring each object was measured. The ORT was performed for the mice fed HFDs for 1 week and orally administered saline or the peptides (3 mg/kg/day).

The OLT was carried out in the experimental procedures similar to the ORT, but in the familiarization session, two identical objects were placed in the adjacent corners of the field and each mouse explored these objects. In the test session, one of the two objects was moved to the opposite corner. Each mouse was again released into the field for 20 s of total exploration, and the time spent exploring the familiar object and the moved object was measured.

### 2.7 Open‐field test (OFT)

The open‐field test (OFT) was performed as previously described.^12^ The apparatus was gray and cylindrical, with a diameter of 60 cm and a height of 50 cm. Each mouse was placed at the center of the arena and allowed to explore for 5 min. We measured the time spent in the center circle and the times of entries to the center field.

### 2.8 BrdU incorporation in the hippocampus

BrdU incorporation was assessed as reported previously.^3^, ^4^, ^13^ The mice in this operation were administered ILPEY (3 mg/kg/day p.o.) starting 1 day after switching to the HFD. On Day 9 after starting administration, BrdU (100 mg/kg) was intraperitoneally administered. After 24 h from BrdU administration, mice were euthanized and transcardially perfused with phosphate‐buffered saline (PBS) followed by 4% paraformaldehyde under anesthesia (*n* = 5–6). Brains were then fixed in 4% paraformaldehyde (4°C for 1 day) and dehydrated in 20% sucrose/PBS (4°C for 3 days). Thin sections of the brains (30 μm/section) were collected on a freezing microtome. Then, they were washed with PBS and incubated in 2 N HCl for 45 min. After incubation, the sections were washed with PBS and blocked in 3% normal donkey serum/PBS with Triton X‐100 (PBST) for 1 h, followed by incubation overnight with anti‐rat BrdU (1:1000; Abcam, Cambridge, UK) at room temperature. Thereafter, they were incubated with a secondary antibody (1:500, donkey anti‐rat Alexa 594; Life Technologies, Carlsbad, CA, USA) for 2 h. BrdU‐positive cells in the hippocampus were counted using an Olympus microscope.

### 2.9 RNA preparation and quantitative RT‐PCR

The hippocampus was excised 2 h after administration as previous study^3^, ^4^ and stored at −80°C until RNA extraction. Total RNA was extracted using the RNeasy Lipid Tissue Kit (QIAGEN Sciences Inc., Osaka, Japan) and transcribed using the Takara PrimeScript RT Master Mix (Takara, Osaka, Japan). For quantitative PCR, we amplified the cDNA using the LightCycler 96 System (Roche Diagnostics Co., Mannheim, Germany) with THUNDERBIRD qPCR Mix (Toyobo Co., Osaka, Japan) and each primer set specific for mouse *brain‐derived neurotrophic factor* (*BDNF*), *nerve growth factor* (*NGF*), *neurotrophin‐3* (*NT‐3*), *glial cell line‐derived neurotrophic factor* (*GDNF*), *epidermal growth factor* (*EGF*), *ciliary neurotrophic factor* (*CNTF*), *fibroblast growth factor 2* (*FGF‐2*), *insulin‐like growth factor 2* (*IGF‐2*), or *vascular endothelial growth factor* (*VEGF*) according to the manufacturer's instructions (Table S1). The reactions were cycled 45 times with denaturation at 95°C for 10 s, and annealing and elongation at 65°C for 60 s. The relative expression level of each mRNA was normalized using the mRNA level of β‐actin.

### 2.10 Oral glucose tolerance test (OGTT) and insulin tolerance test (ITT)

The glucose‐lowering effect was assessed using the oral glucose tolerance test (OGTT). The OGTT was performed as described previously.^3^, ^4^, ^14^ Briefly, glucose solution was administered orally at a dose of 2 g/kg to mice fasted for 18 h on Day 7. Saline or ILPEY was administered on Day 7, 2 h before glucose administration. Blood was obtained from the tail vein. The blood glucose levels were immediately measured before, and 15, 30, 60, and 90 min after glucose administration using the One Touch Ultra View (LifeScan Japan Corp., Tokyo Japan). The ITT was also performed as reported previously.^3^, ^14^ Briefly, insulin dissolved in saline was intraperitoneally administered (1.0 U/kg) to mice fasted for 5 h on Day 7. The tail vein blood glucose levels were measured. Saline or ILPEY was administered p.o., 2 h before insulin administration. The tail vein blood glucose levels were measured before, and 15, 30, 60, and 90 min after insulin administration. The OGTT and ITT were started at 11:00 a.m., during the light phase of the light/dark cycle.

### 2.11 Statistical analysis

All values are expressed as the means ± SEM. Analysis of variance followed by Student's *t*‐test and Dunnett's test were used to assess differences among two and three or more groups, respectively. *p*‐values less than 0.05 were considered significant.

## 3 RESULTS

### 3.1 Thermolysin digest of ovalbumin increased cognitive function in mice fed HFDs

**FIGURE 1:**
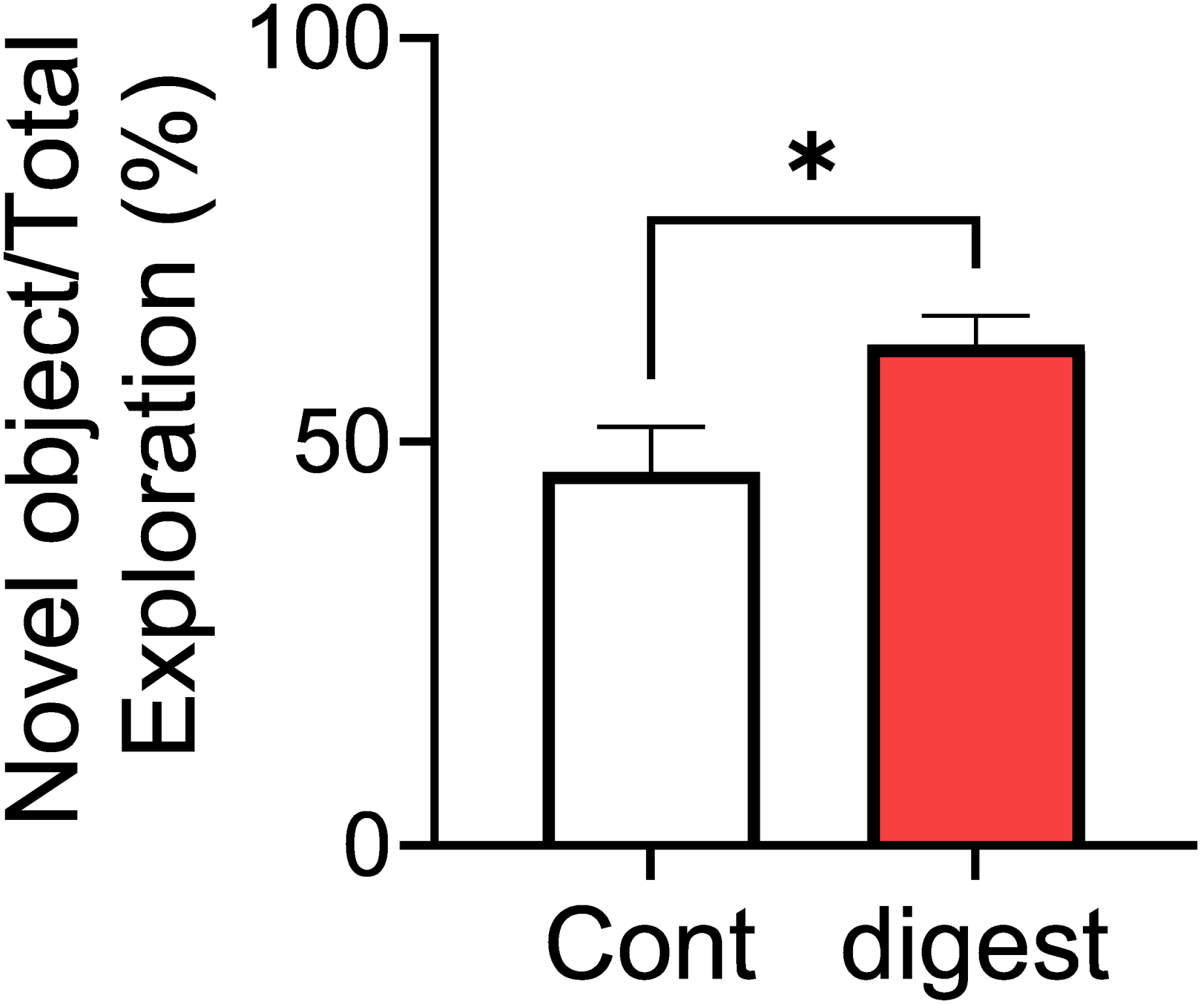
Thermolysin digest of ovalbumin improved cognitive decline induced by HFD intake. Orally administered digest at a dose of 30 mg/kg (once a day for 3 days) in mice fed HFDs for 1 week. Thereafter, the novel ORT was performed. Each value is the mean ± SEM (*n* = 8). *p < 0.05 versus the control group.

The thermolysin digest of ovalbumin (30 mg/kg/day, once a day for 3 days) increased the approach time to novel objects in the ORT after oral administration in mice treated with the HFD for 1 week (Figure 1). This result suggests that the thermolysin digest of ovalbumin improved cognitive function.

### 3.2 Novel cognitive function‐improving peptide, ovomemolin A (OMA) found by predicting the cleavage site of ovalbumin by substrate specificity of thermolysin

**FIGURE 2:**
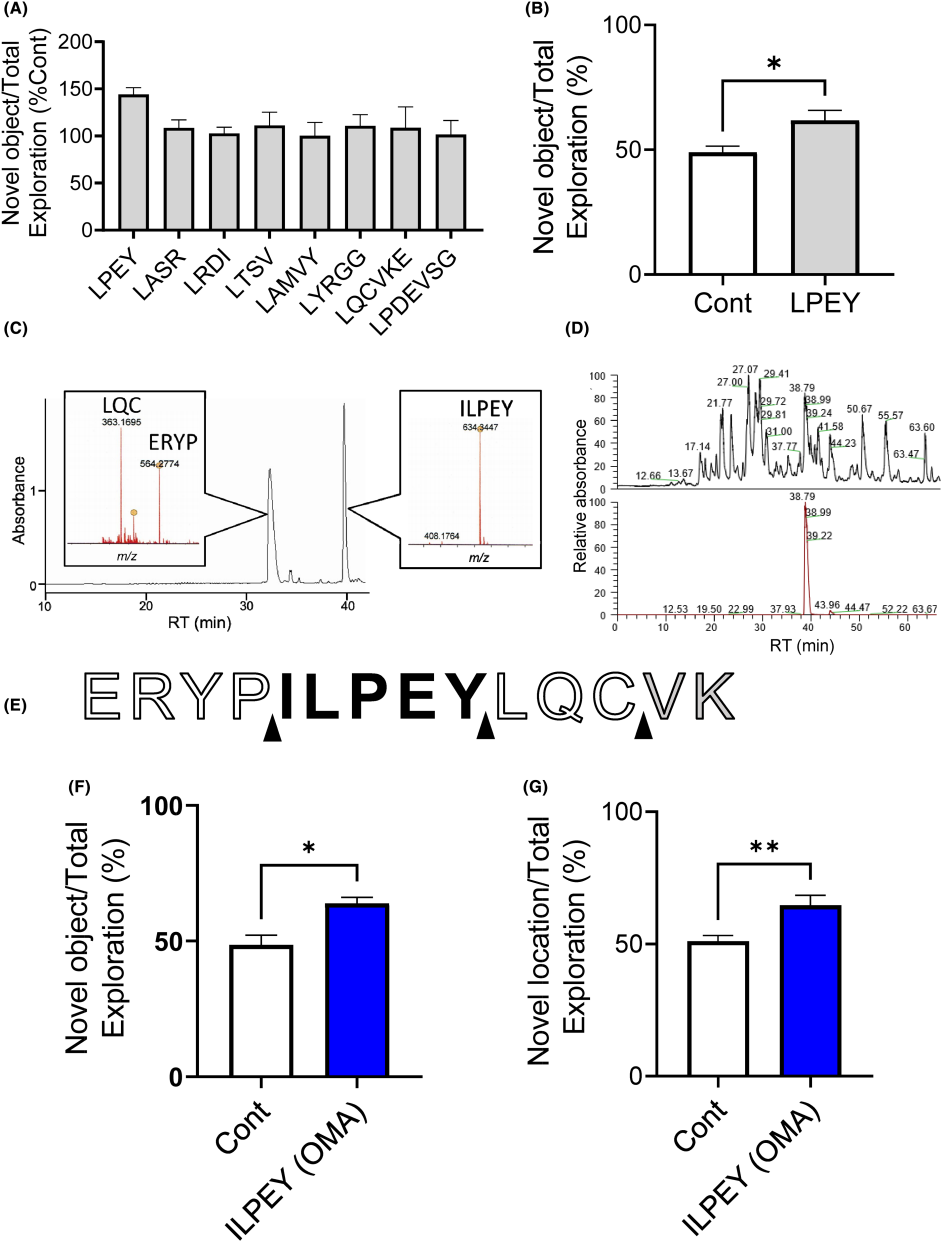
Discovery of ovomemolin A (OMA) based on a prediction of peptide formation by substrate specificity of thermolysin. (A) The comparison of peptide candidates (3 mg/kg/day for 3 days) in the ORT. Values are expressed relative to the percentage of approach time to the novel object in the control group, which was 100. (B) Effect of LPEY on cognitive function in the ORT. (C) HPLC chart of thermolysin digest of model peptide after thermolysin digest and peptide identification by mass spectrometry of each peak (inserts). (D) The chromatogram of thermolysin digest of ovalbumin (upper chart) and the extraction for the isotopic mass of OMA (ILPEY, theoretical m/z = 633.326) (lower chart). (E) The cleavage site of ovalbumin after thermolysin treatment as predicted by model peptide experiment. (F) Orally administered OMA (ILPEY) at a dose of 3 mg/kg/day (once a day for 3 days) in mice fed with HFD, and the ORT was performed. (G) The OLT was performed similarly. All values are the mean ± SEM (A, *n* = 3–4; B, *n* = 5–6; F, *n* = 5–6; G, *n* = 10). *p < 0.05, **p < 0.01 versus the control group.

Next, we searched for cognitive function‐improving peptides in the digest using two strategies: (1) prediction of cleavage sites of ovalbumin based on the substrate specificity of thermolysin, and (2) comprehensive peptide analysis of the digest. Our previous report indicates that thermolysin is easily cleaved on the N‐terminal side of leucine residues.^7^ As shown in Figure S1, we narrowed the candidates down to eight from the peptides predicted based on the substrate specificity. Then, we chemically synthesized these peptides and evaluated whether they can improve cognitive function. In the ORT, we found that LPEY showed the most potent activity (Figure 2A). We also confirmed the effect of LPEY and found a significant increase in the approach time to novel objects in the ORT (Figure 2B). These results suggest that the tetrapeptide improves cognitive function.

We also tested whether this predicted tetrapeptide candidate is released by thermolysin digestion using a model peptide mimicking ovalbumin, extended at both the N‐ and C‐termini with five residues (ERYPILPEYLQCVK). Unexpectedly, a pentapeptide (ILPEY) but not a tetrapeptide (LPEY) was released from the model peptide (Figure 2C). Furthermore, we confirmed that ILPEY is present in the thermolysin digest of ovalbumin (Figure 2D,E). We then tested the effect of the ILPEY on cognitive function.

At a dose of 3 mg/kg/day, ILPEY increased the approach time to novel objects in the ORT after oral administration (Figure 2F). In the OLT, the ILPEY increased the approach time to the object in a new location (Figure 2G). Thus, we demonstrated that orally administered ILPEY improved cognitive decline induced by HFD intake in two different paradigms, and we named the peptide OMA.

### 3.3 Ovomemolin B (OMB) and C (OMC) found by comprehensive peptide analysis of the ovalbumin digest

**FIGURE 3:**
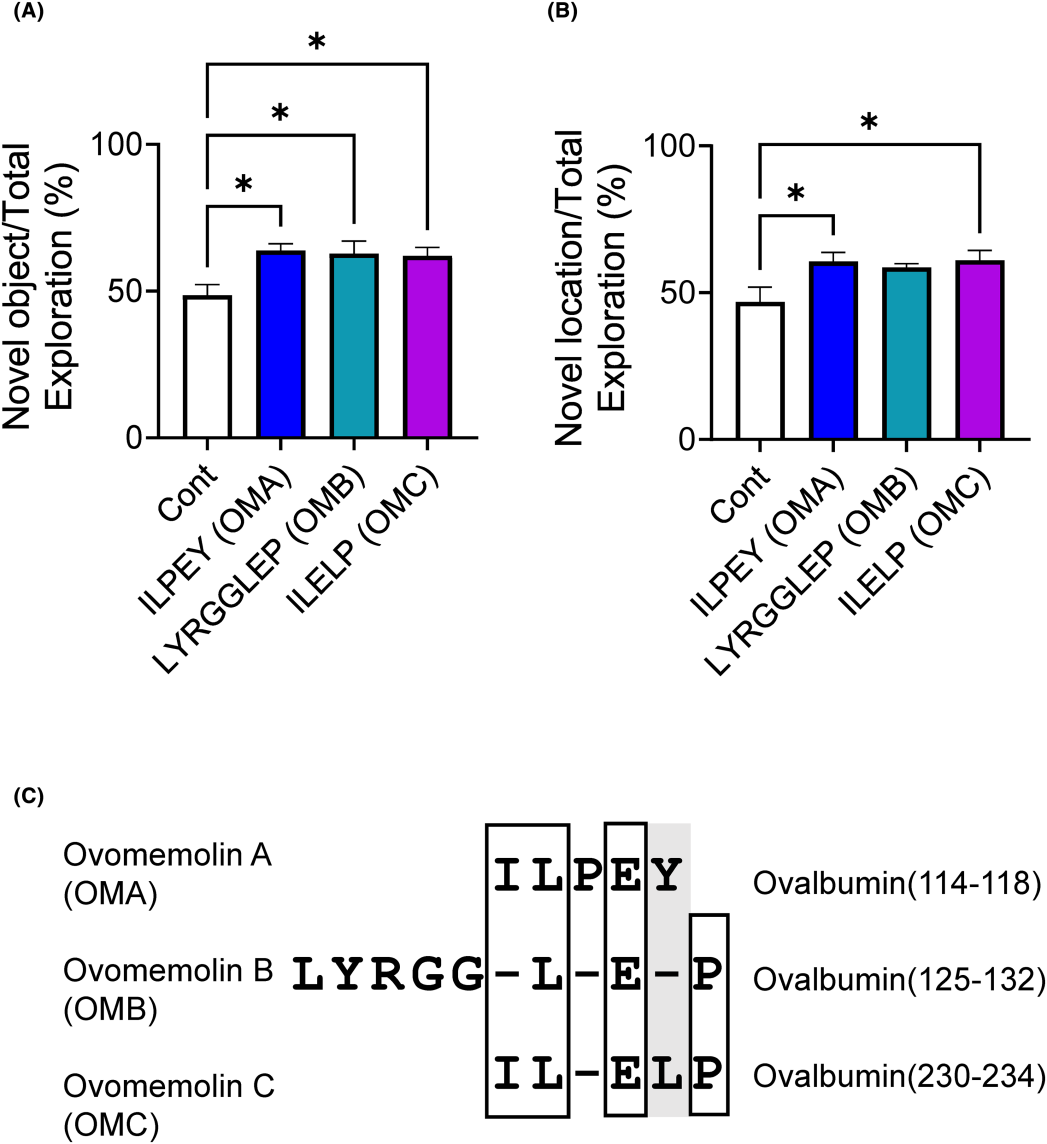
Discovery of OMB and OMC based on comprehensive peptide analysis of the thermolysin digest, and comparison with OMA. (A) ILPEY, LYRGGLEP, and ILELP (OMA, OMB, and OMC, respectively) were administered at a dose of 3 mg/kg/day (once a day for 3 days) in mice fed with HFD, and thereafter, the ORT was performed. (B) The OLT was performed under the same condition. All values are the mean ± SEM. (A and B, *n* = 4–5) *p < 0.05 versus the control group. (C) Homology of ovomemolins. The solid line indicates the same amino acid residues, and the gray block indicates a similar sequence.

In addition, a comprehensive peptide analysis of the ovalbumin digest (Table 1) was used to identify peptides. We tested whether the top five candidates with the largest peak area in this analysis improved cognitive function. The top two peptides, LYRGGLEP and ILELP, improved cognitive decline in the ORT after oral administration, similar to ILPEY (each 3 mg/kg/day, once a day for 3 days) (Figure 3A). In the OLT, ILELP increased the approach time to the object in a new location (3 mg/kg/day) (Figure 3B). LYRGGLEP also appeared to increase the mean of the approach time, similar to that of ILPEY. In contrast, the top 3–5 peptides (each 3 mg/kg/day) did not increase the approach time to novel objects in the ORT (data not shown). In addition, LYRGGLEP and ILELP were demonstrated to be present in the thermolysin digest of the ovalbumin by LC–MS (Table 2). Thus, LYRGGLEP and ILELP were termed OMB and OMC, respectively. Notably, there is homology between OMA, OMB, and OMC (Figure 3C), and this common structure may contribute to the improvement of cognitive function. Next, we focused on OMA because of its relatively high content (Table 2).

**TABLE 1:** Comprehensive peptide analysis for thermolysin digest of ovalbumin.

Sequence	Theoretical *m*/*z*, [M + H]+	Average retention time (min)	*m*/*z* detected (Da)	Charge	Xcorr^a^	Peak area
LYRGGLEP	452.748	27.2	452.748	2	1.89	2.12E+09
ILELP	584.366	55.5	584.366	1	1.39	1,985,624,576
LYAEERYP	1040.505	28.7	1040.505	1	2.71	1,919,523,145
ITKPNDVYS	518.770	21.4	518.770	2	1.42	1,900,419,584
FDKLPG	338.687	23.5	338.687	2	1.81	1,699,024,636
FKDEDTQAMP	1181.515	26.9	1181.515	1	2.12	1,465,020,909
LINSWVESQTNG	674.331	50.8	674.331	2	2.36	1,314,689,243
LINSW	632.341	44.0	632.341	1	1.61	1,263,672,192
LRDILNQ	436.254	29.6	436.254	2	2.06	1,097,449,343
LVLLPDEVSG	521.296	63.7	521.296	2	1.74	1,042,433,492

^a^ XCorr, the cross‐correlation value between theoretical MS2 fragments deduced from peptide sequence and measured MS2 spectrum.^15^ When the XCorr was below 0.5, unreliable peptides were omitted.

**TABLE 2:** Ovomemolin concentrations in the ovalbumin digest.

Peptide		Concentration (ug/mg)
ILPEY	Ovomemolin A	11.0
LYRGGLEP	Ovomemolin B	9.0
ILELP	Ovomemolin C	2.9

### 3.4 Characterization of orally active ovomemolin A (OMA)

**FIGURE 4:**
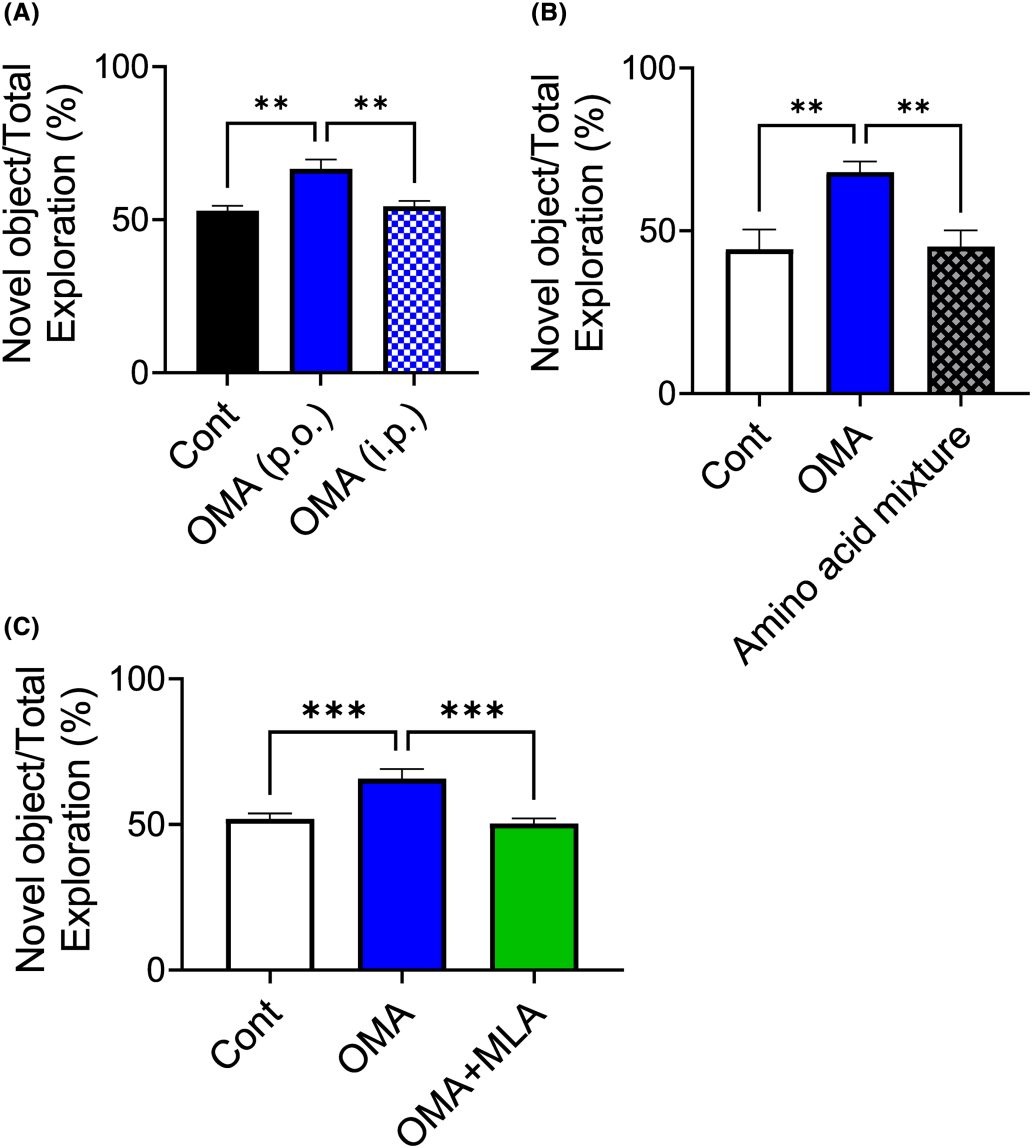
Characterization of OMA. (A) Effect of administration routes on the cognitive function. OMA was administered orally or intraperitoneally (i.p.) at a dose of 3 mg/kg/day (once a day for 3 days) in mice treated with HFD, and the ORT was performed. (B) Comparison of OMA's constituent amino acid mixture with OMA itself. OMA peptide (3 mg/kg/day for 3 days) and amino acid mixture equivalent to the OMA were administered orally, and the ORT was performed. (C) Effect of α7 nicotinic acetylcholine receptor antagonist on the OMA‐induced cognitive decline improvement. OMA (3 mg/kg/day for 3 days) and MLA (0.3 mg/kg/day for 3 days) were administered orally, and the ORT was performed. Values are the mean ± SEM (A, *n* = 4–7, B, *n* = 5–6; C, *n* = 7–9). **p < 0.01, ***p < 0.001 as compared with each group.

To identify the mechanism of ILPEY action, we orally and intraperitoneally administered OMA. Orally but not intraperitoneally administered OMA increased the approach time to the novel object in the ORT (Figure 4A). This result suggests that OMA may be acting on the gastrointestinal tract rather than being absorbed.

To investigate whether the mechanism of OMA depends on its sequence rather than amino acids resulting from the peptide degradation, we investigated the effect of an amino acid mixture composed of OMA. OMA but not the amino acid mixture (equivalent to OMA, 3 mg/kg/day) increased the approach time to a novel object, suggesting that the OMA sequence is important for cognitive function improvement (Figure 4B).

### 3.5 The cognitive improvement of OMA was coupled to the acetylcholine system

Acetylcholine (ACh) is a significant neurotransmitter that plays important roles in memory and learning.^16^, ^17^ The α7 nicotinic acetylcholine receptor (α7nAChR) is a subunit of the ACh receptor and a promising therapeutic target in neurodegenerative diseases.^18^ We thus investigated the pathway of the ACh system using methyllycaconitine (MLA), an antagonist of α7nAChR. MLA suppressed the OMA‐induced increase in the approach time to novel objects in the ORT (OMA, 3 mg/kg/day and MLA, 0.3 mg/kg/day, once a day for 3 days). These results suggest that orally administered OMA improves cognitive function associated with the ACh system (Figure 4C).

### 3.6 Effect of OMA on the hippocampal neurogenesis and mRNA expression

**FIGURE 5:**
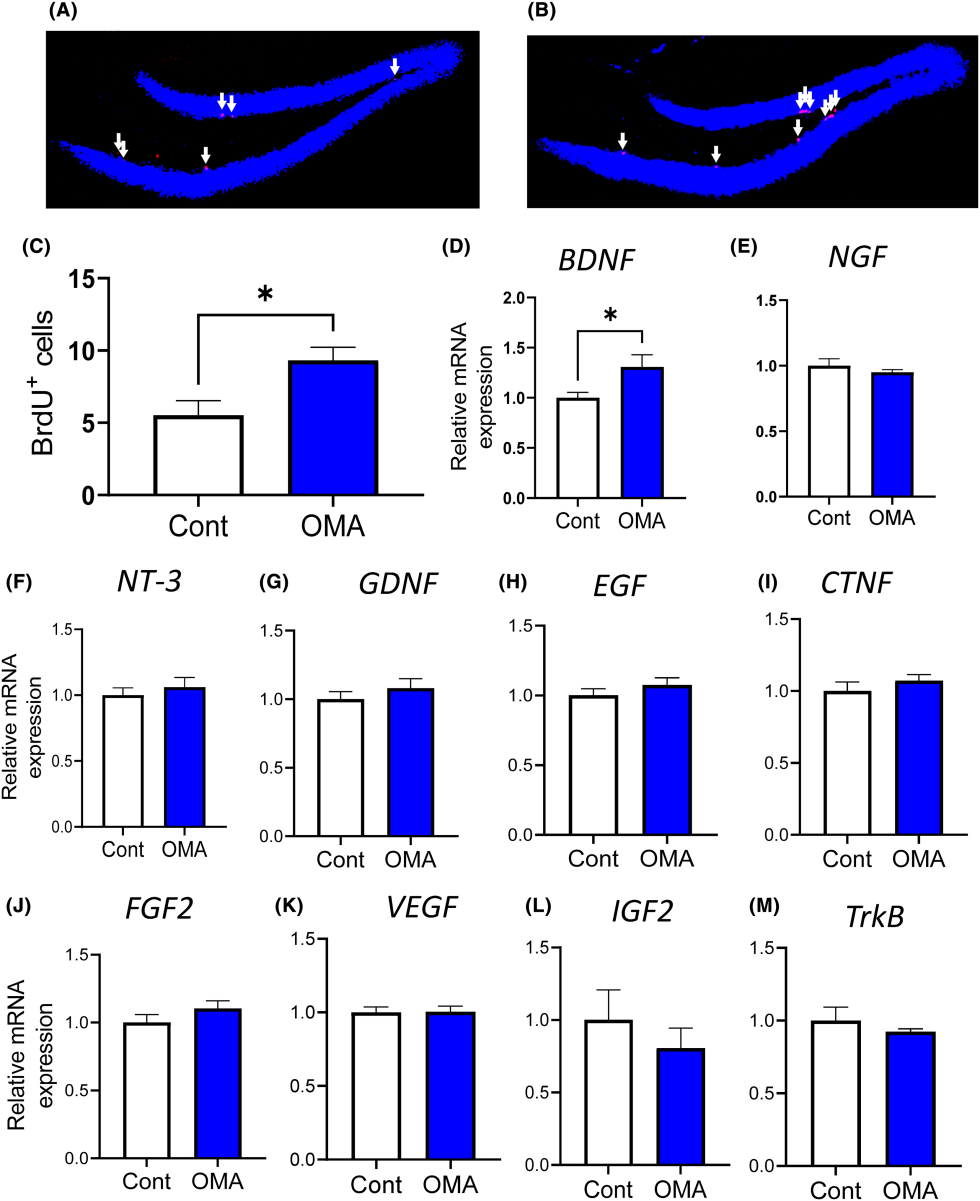
Changes in the hippocampus after OMA administration. (A–C) OMA increases the BrdU‐positive cells in the hippocampus. (A,B) Representative histology of the control group (A) and OMA group (B). White arrows indicate BrdU‐positive cells. (C) Number of BrdU‐positive cells. (D–M) Changes in the hippocampal mRNA expression related to neurotrophic factors. The following neurotrophic factors were measured by quantitative RT‐PCR: *BDNF* (D), *NGF* (E), *NT‐3* (F), *GDNF* (G), *EGF* (H), *CNTF* (I), *FGF‐2* (J), *IGF‐2* (K), *VEGF* (L), and *TrkB* (M). All values are the mean ± SEM (C–M, *n* = 5–6). *p < 0.05 versus the control group.

It has been reported that hippocampal neurogenesis relates to cognitive improvement.^19^, ^20^, ^21^ We also previously revealed that BrdU‐positive cells increased in the mice treated with cognitive decline‐improving peptides.^3^ We therefore examined the BrdU‐positive cells in the hippocampus (Figure 5A–C). The OMA increased the number of BrdU‐positive cells in the hippocampal dentate gyrus compared with the control mice after oral administration, implying that OMA increases hippocampal neurogenesis.

Neurotrophic factors influence neural survival and have been reported to be associated with cognitive function.^22^ We then measured the hippocampal mRNA expression of several neurotrophic factors: *BDNF*, *NGF*, *NT‐3*, *GDNF*, *EGF*, *CNTF*, *FGF‐2*, *IGF‐2*, and *VEGF* (Figure 5D–L). Among these genes, a significant increase in *BDNF* expression was observed after OMA administration (Figure 5D). *BDNF* has been reported to have an important role in the promotion of neurogenesis and improving cognitive function.^23^ Therefore, these results suggest that OMA increases the hippocampal expression of *BDNF*, which subsequently affects neurogenesis and cognitive function. Changes in mRNA expression of *TrkB*, the receptor for BDNF, were not observed (Figure 5M). It was revealed that ACh is important in the cognitive‐improving function of OMA (Figure 4C); however, changes in mRNA expression of ACh‐related factors (*ChAT* and *AChE*, the factors important for ACh synthesis, and *α7nAChR*) were not observed (Figure S2A‐C) Additionally, changes in the expressions of inflammatory cytokines (*TNF‐α* and *IL‐1β*, and endoplasmic reticulum stress; *ATF‐4* and *CHOP*) were not observed (Figure S2D‐G).

## 4 DISCUSSION

We found that the thermolysin digest of ovalbumin, a major protein derived from egg whites, improves cognitive decline induced by HFD intake after oral administration. Candidate peptides that improve cognitive decline were identified using two strategies: peptide release prediction based on the substrate specificity of the enzyme and comprehensive analysis of the digest. Finally, we found three novel and orally active peptides, ILPEY, LYRGGLPE, and ILELP. These peptides were demonstrated to be present in the digest, and we named them OMA, OMB, and OMC, respectively.

We further examined the peptide with the highest content in the digest, OMA. Oral administration of OMA was effective but intraperitoneally administration was not. Assuming that OMA is absorbed from the gastrointestinal tract and reaches the brain to exert its effect, it is also expected to exert its effect after intraperitoneally administration; however, intraperitoneally administered OMA was ineffective. Additionally, a mixture of constituent amino acids of OMA was ineffective. Although the ovomemolins are considered unfavorable for absorption owing to their relatively large molecular weight, they all exert their effects when administered orally. Thus, we hypothesize that orally administered ovomemolins act on the gastrointestinal tract directly, and the signal is transferred to the brain. We have reported several cases in which medium molecular weight peptides acting by oral administration are mediated through gut–brain communication.^7^ We digested OMA using pepsin and pancreatin, mimicking enzymatic digestion in the gastrointestinal tract, and analyzed them by LC–MS. OMA was detected in the pepsin digest but not in the pepsin‐pancreatin digest (Figure S3). These suggest that OMA acts in the stomach or upper intestine, and its signals are transmitted to the brain. In addition, OMB and OMC were detected in both pepsin and pepsin‐pancreatin digests, indicating their enzymatic digestion resistance in the digestive tract (Figure S3).

We found that orally administered OMA improved cognitive decline through the ACh system, the most important mediator associated with memory and learning. The OMA‐induced improvement of cognitive function was blocked by peripheral administration of an antagonist for the α7nAChR, implying that not only central but also peripheral α7nAChR is involved in the cognitive improvement. Activation of α7nAChR on the gastrointestinal tract has been shown to improve cognitive function.^24^ The receptor expression is predominantly in the central nervous system, but it is also present in the stomach and small intestine,^25^ in the large intestine, and in intestinal epithelial cells, glial cells, and macrophages.^26^ Further study may elucidate how OMA activates the acetylcholine‐α7nAChR system.

In addition, OMA increases the mRNA expression of hippocampal *BDNF* and promotes BrdU‐positive cells after oral administration, suggesting that this neurotrophic factor induces enhanced hippocampal neurogenesis. BDNF is a neurotrophic factor that acts on neuroprotection and synaptogenesis and is closely related to learning and memory.^23^ It also acts on cholinergic neurons in the central nervous system and has been the target of drug discovery research for neurodegenerative diseases such as Alzheimer's disease and Parkinson's disease.^16^, ^17^ The peptides we previously found that improve cognitive decline induced by HFD intake increase other neurotrophic factors in the hippocampus; YLG, derived from α_S1_‐casein, increases *NGF* and *CTNF*, and rice‐memolin, derived from rice bran, increases *EGF* and *FGF‐2*, and slightly increase *NGF*, *NT‐3*, and *GDNF*. This difference in the increasing neurotrophic factors suggests that OMA may improve cognitive function through a different pathway from these peptides. The α7nAChR‐dependent *BDNF* upregulation was previously reported, and our results on OMA were consistent with this pathway.

It is also known that cognitive function is affected by glucose metabolism. We previously reported that milk‐derived YLG used to improve cognitive decline induced by HFD intake increased insulin sensitivity in the ITT.^3^ VYTPG, named rice‐memolin, also improved glucose metabolism after oral administration.^4^ In contrast, OMA improved cognitive function but did not affect blood glucose levels in the OGTT and ITT (Figure S4). Furthermore, OMA does not have a clear homology with YLG and VYTPG, implying that OMA may act through a different pathway.

Several peptide formations can be predicted after enzymatic digestion based on the substrate specificity of the enzyme; however, the accuracy of the prediction is limited, and substrate specificities other than trypsin are not as rigorous. Thus, experimental quantification of candidate peptides is essential for identification. Comprehensive peptide analysis has been used for identifying anxiolytic‐ or antidepressant‐like peptides and appetite‐regulating peptides.^7^, ^27^, ^28^ In the present study, two novel peptides, OMB and OMC, were discovered by applying this technique. Furthermore, using the new structure–activity relationship information obtained in this study, novel peptides for improving cognitive function may be discovered based on the comprehensive analysis of other protein digests. OMA, OMB, and OMC exhibit cognitive improvement at a dose of 3 mg/kg/day. All OMs showed no significant effect at a dose of 1 mg/kg/day, so their minimum effective doses are estimated to be 3 mg/kg/day. Thus, we cannot rule out the possibility that the effects of bioactive peptides other than OMA, OMB, and OMC may contribute to the improvement of cognitive function by the digest.

Overall, chicken eggs contain a variety of nutrients, such as high‐quality protein, vitamins, and minerals, which have been reported to have a variety of health benefits. In this study, we added ovomemolins to the list of bioactive substances in eggs. In addition, food‐derived peptides can be used easily and safely and potentially incorporated into daily lives as a functional food to prevent cognitive impairment at an early stage. Various food‐derived ingredients have been shown to improve cognition, dependent of various mechanisms.^29^, ^30^, ^31^ It would be ideal if health maintenance could be achieved through the regular intake of easily available foods.

The other limitations of this study are still existing. Molecules, cells, and tissues targeted by orally active peptides, their bioavailability, metabolism, and absorption in vivo, the gut–brain communication, time course of changes in hippocampal expression of genes related to memory and translation of the mouse models to human clinical studies should be clarified in future.

In summary, we revealed the cognitive‐improving effect of the enzymatic digest of ovalbumin after oral administration. We specifically found three peptides in the digest that improve cognitive decline induced by HFD intake. These peptides were termed ovomemolins. To the best of our knowledge, ovomemolins are the first egg‐derived peptides reported to improve cognitive decline after oral administration.

## AUTHOR CONTRIBUTIONS

Kousaku Ohinata supervised and designed the experiments. Takanobu Nakajima, with help from Maiko Shobako and Kentaro Kaneko, performed the experiments and analyzed the data. Maiko Shobako conducted preliminary studies that led to the discovery of OMs. Atsushi Kurabayashi and Maiko Shobako performed comprehensive peptide analysis by LC–MS/MS. Takanobu Nakajima, Maiko Shobako, and Kousaku Ohinata wrote the paper. All authors discussed the results and the manuscript.

## CONFLICT OF INTEREST STATEMENT

The authors declare no conflicts of interest related to the content of this paper.

## Supporting information


**Figure S1:** Selection of peptide candidates derived from thermolysin digestion of ovalbumin. (A) The number of amino acids at the N‐terminus in comprehensive peptide analysis of thermolysin digest of soy β‐conglycinin (Mori et al., FASEB J. 2018;32(2):568–575.). Thermolysin often cleaved the N‐terminal side of Leu residues of protein. (B) The peptide candidates we selected in the ovalbumin sequence. Leu residues are highlighted. Bold and underlined sequences are the ones. Each value is the mean ± SEM (*n* = 6).
**Figure S2:** The hippocampal mRNA expression after ovomemolin A (OMA) administration. (A–C) Acetylcholine (ACh)‐associated factors. (D–E) inflammatory cytokines. (F–G) Endoplasmic reticulum stress factors.
**Figure S3:** LC–MS analysis of OMs after pepsin and pancreatin digestion to imitate the digestion in the gastrointestinal tract. Each enzyme treatment time was 5 h. The UHPLC–MS system was consisted of Ultimate 3000 RSLC liquid chromatograph equipped with a Presto FF‐C18 column (Imtakt, Kyoto, Japan), and Q Exactive mass spectrometer (Thermo Fisher Scientific, Waltham, MS, USA). The peptides were separated with a linear gradient elution of 0.1% formic acid in water and 0.1% formic acid in acetonitrile over 45 min.
**Figure S4:** (A, B) Blood glucose levels measured using the ITT with the area under the curve (AUC) calculated. (C, D) Blood glucose levels measured using the OGTT with the AUC calculated . (E, F) Time spent in the center circle (E) and entry to the center circle measured in the open filed test. Each value is the mean ± SEM (A, B, *n* = 5–6; C, D, *n* = 10–11; E, F, *n* = 8‐9).

**Table S1.** Primer sets used for quantitative RT‐PCR.

## Data Availability

Stored in repository.
