# Supplementary material for: Ovomemolins: Egg‐derived peptides that improved cognitive decline after oral administration in mice
Source: FASEB Bioadv. 2024 Jun 10;6(7):177–88. doi: 10.1096/fba.2023-00149 (PMC11226991; doi:10.1096/fba.2023-00149)
Supplement: Supplementary file 1 — Figure S1: Selection of peptide candidates derived from thermolysin digestion of ovalbumin. (A) The number of amino acids at the N‐terminus in comprehensive peptide analysis of thermolysin digest of soy β‐conglycinin (Mori et al., FASEB J. 2018;32(2):568–575.). Thermolysin often cleaved the N‐terminal side of Leu residues of protein. (B) The peptide candidates we selected in the ovalbumin sequence. Leu residues are highlighted. Bold and underlined sequences are the ones. Each value is the mean ± SEM (n = 6). Figure S2: The hippocampal mRNA expression after ovomemolin A (OMA) administration. (A–C) Acetylcholine (ACh)‐associated factors. (D–E) inflammatory cytokines. (F–G) Endoplasmic reticulum stress factors. Figure S3: LC–MS analysis of OMs after pepsin and pancreatin digestion to imitate the digestion in the gastrointestinal tract. Each enzyme treatment time was 5 h. The UHPLC–MS system was consisted of Ultimate 3000 RSLC liquid chromatograph equipped with a Presto FF‐C18 column (Imtakt, Kyoto, Japan), and Q Exactive mass spectrometer (Thermo Fisher Scientific, Waltham, MS, USA). The peptides were separated with a linear gradient elution of 0.1% formic acid in water and 0.1% formic acid in acetonitrile over 45 min. Figure S4: (A, B) Blood glucose levels measured using the ITT with the area under the curve (AUC) calculated. (C, D) Blood glucose levels measured using the OGTT with the AUC calculated . (E, F) Time spent in the center circle (E) and entry to the center circle measured in the open filed test. Each value is the mean ± SEM (A, B, n = 5–6; C, D, n = 10–11; E, F, n = 8‐9). [file FBA2-6-177-s001.pdf]

A

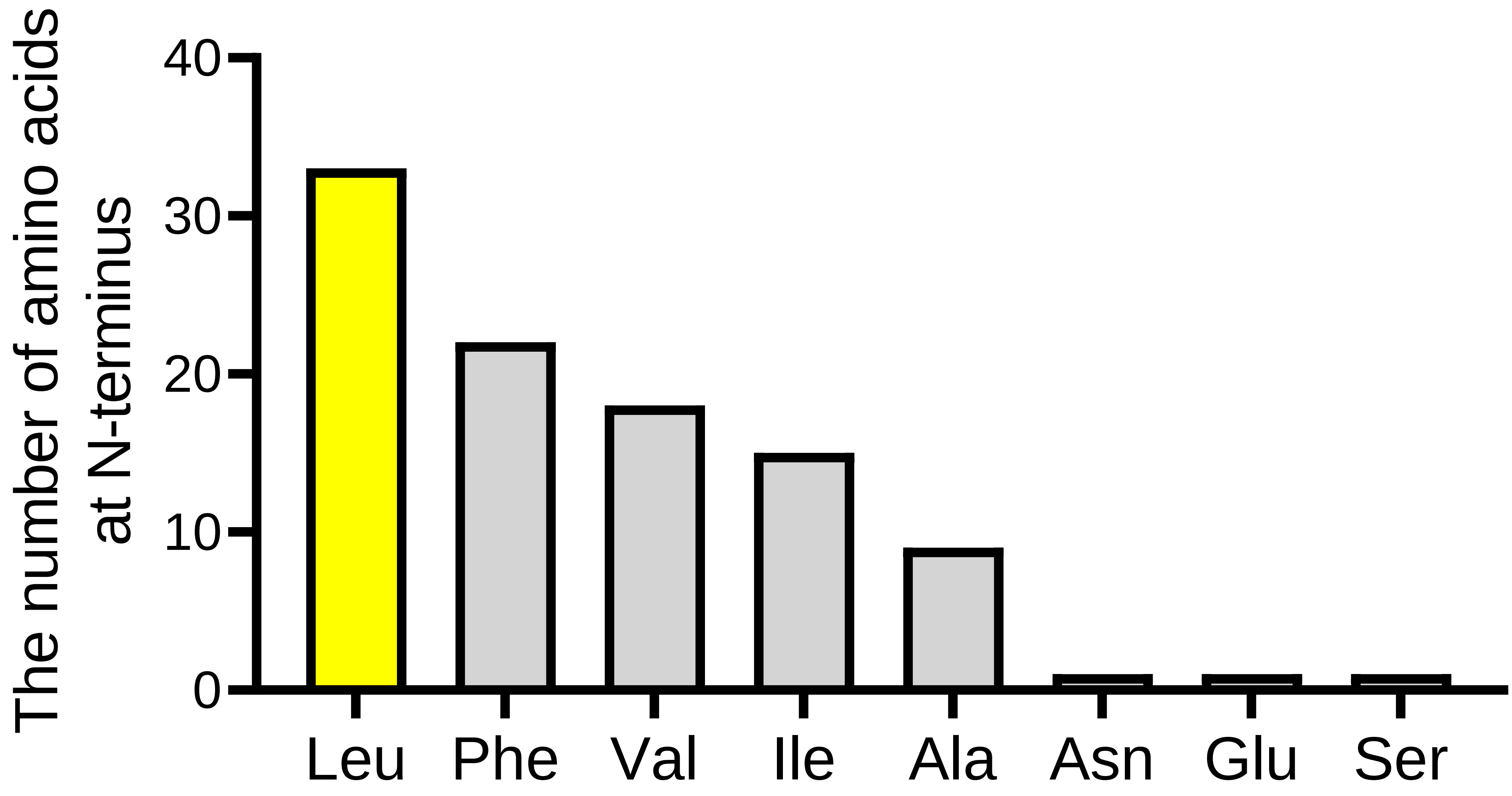

B

MGSIGAASMEFCFDVFKELKVHHANENIFYCPIAIMSA
LAMVYLGAKDSTRTQINKVVRFDKLPGFGDSIEAQCGT
SVNVHSS**LRDI**LNQITKPNDVYSFS**LASR**LYAEERYPI
LPEYLQCVKELYRGGLEPINFQTAADQARELINSWVES
QTNGIIRNVLQPSSVDSQTAMVLVNAIVFKGLWEKAFK
DEDTQAMPFRVTEQESKPVQMMYQIGLFRVASMASEKM
KILELPFASGTMSMLVL**LPDEVSG**LEQLESIINFEKLT
EWTSSNVMEERKIKVYLPRMKMEEKYN**LTSV**LMAMGIT
DVFSSSANLSGISSAESLKISQAVHAAHAEINEAGREV
VGSAEAGVDAASVSEEFRADHPFLFCIKHIATNAVLFF
GRCVSP

Supplemental Figure 1

Selection of peptide candidates derived from thermolysin digestion of ovalbumin.
A) The number of amino acids at the N-terminus in comprehensive peptide analysis of thermolysin digest of soy β-conglycinin (Mori *et al.*, FASEB J. 2018 Feb;32(2):568-575.). Thermolysin often cleaved the N-terminal side of Leu residues of protein. (B) The peptide candidates we selected in the ovalbumin sequence. Leu residues are highlighted. Bold and underlined sequences are the ones.

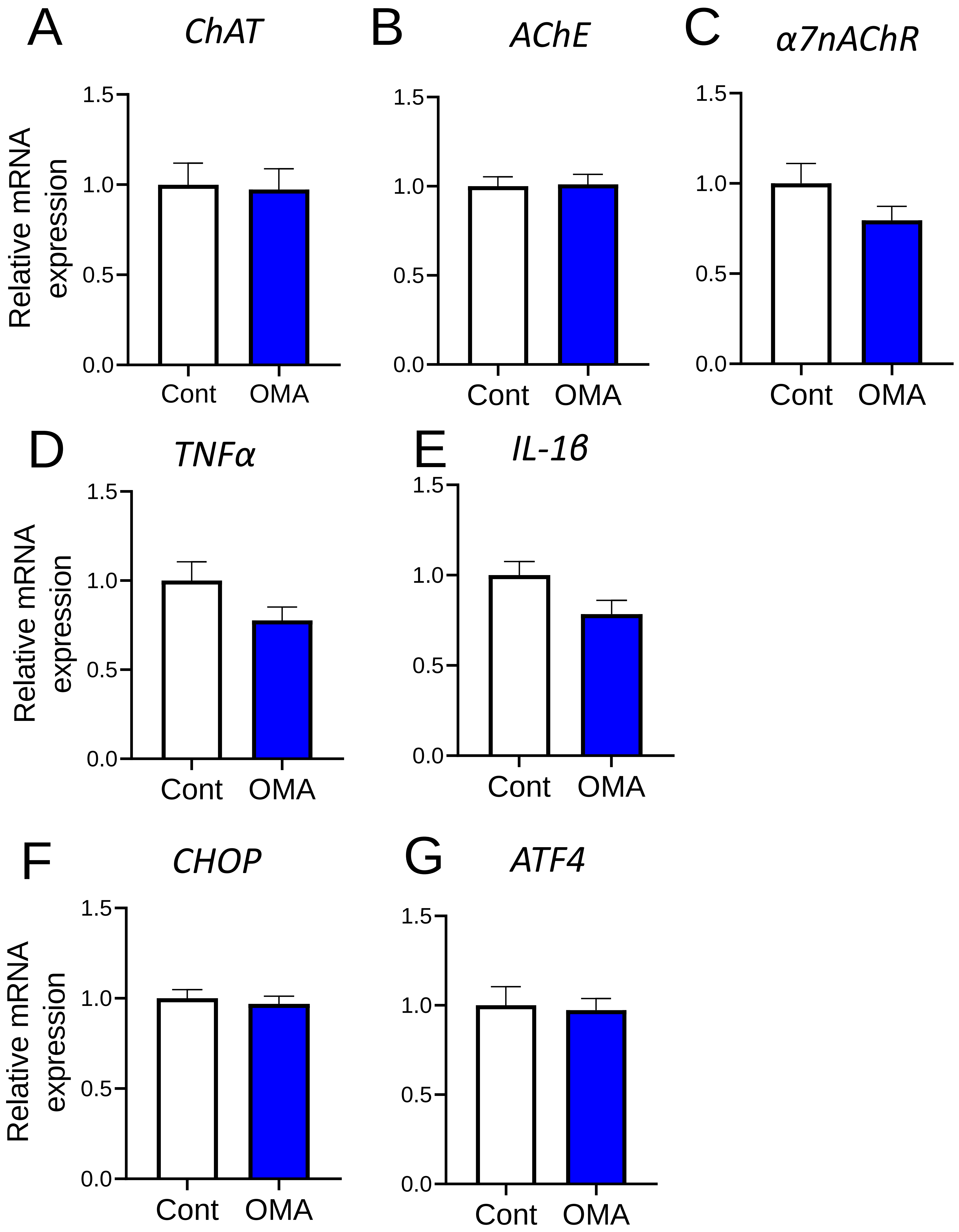

Supplemental Figure 2

The hippocampal mRNA expression after ovomemolin A (OMA) administration. A-C) acetylcholine (Ach)-associated factors. D-E) inflammatory cytokines. F-G) endoplasmic reticulum stress factors. Each value is the mean ± SEM (n = 6).

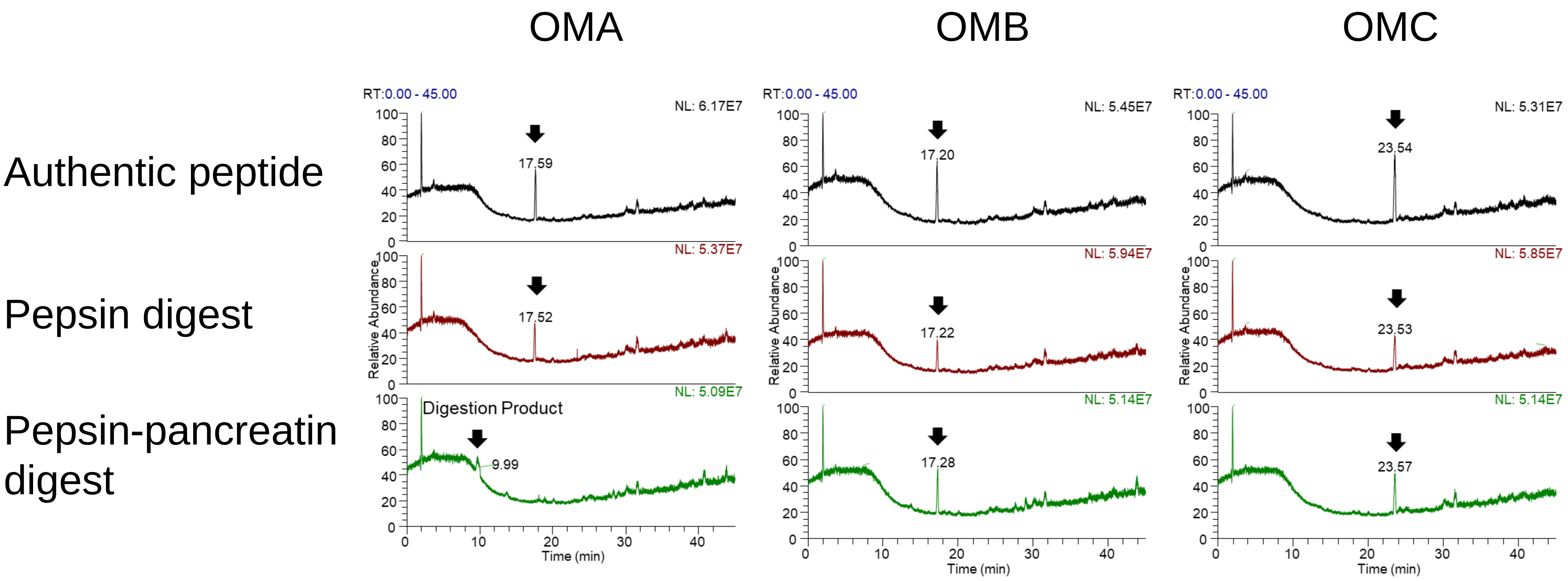

Supplemental Figure 3

LC-MS analysis of OMs after pepsin and pancreatin digestion to imitate the digestion in the gastrointestinal tract. Each enzyme treatment time was 5 h. The UHPLC-MS system was consisted of Ultimate 3000 RSLC liquid chromatograph equipped with a Presto FF-C18 column (Imtakt, Kyoto, Japan), and Q Exactive mass spectrometer (Thermo Fisher Scientific, Waltham, MS, USA). The peptides were separated with a linear gradient elution of 0.1% formic acid in water and 0.1% formic acid in acetonitrile over 45 min.

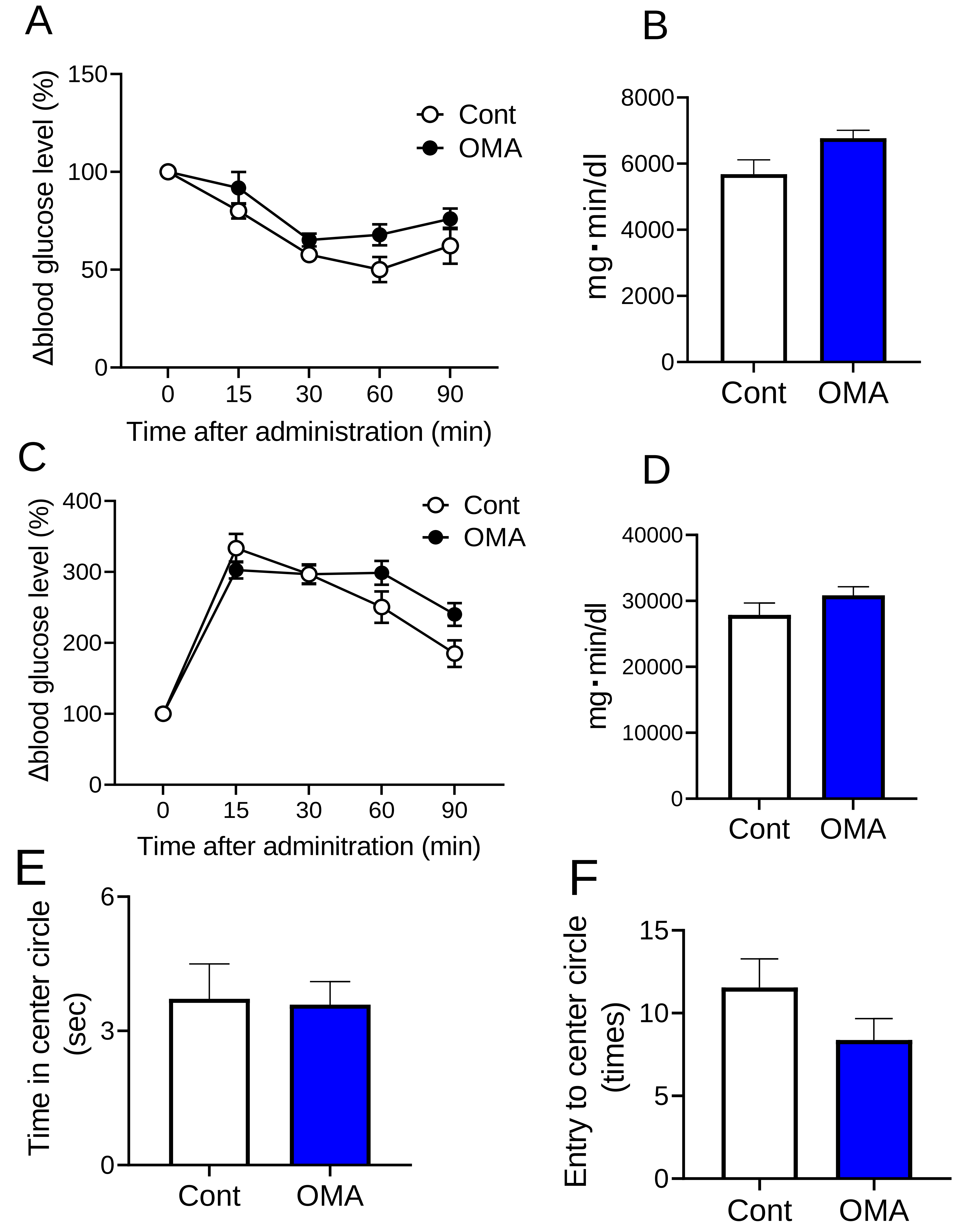

Supplemental Figure 4

A, B) Blood glucose levels measured using the ITT with the area under the curve (AUC) calculated. C, D) Blood glucose levels measured using the OGTT with the AUC calculated. E, F) Time spent in the center circle and entry to the center circle measured in the open filed test. Each value is the mean ± SEM (A, B, *n* = 7–8; C, D, *n* = 6–7; E, F, *n* = 8-9).
