# Supplementary material for: Ovomemolins: Egg‐derived peptides that improved cognitive decline after oral administration in mice
Source: FASEB Bioadv. 2024 Jun 10;6(7):177–88. doi: 10.1096/fba.2023-00149 (PMC11226991; doi:10.1096/fba.2023-00149)
Supplement: Supplementary file 2 — Table S1. Primer sets used for quantitative RT‐PCR. [file FBA2-6-177-s002.pdf]

Supplemental Table 1. Primer sets used for quantitative RT-PCR

Genes	Reverse	Forward
BDNF	5’-TCAGTTGGCCTTTGGATACC-3’	5’-GCGGCAGATAAAAAGACTGC-3’
NGF	5’-CTGTGTCAAGGGAATGCTGA-3’	5’-CAGGCAGAACCGTACACAGA-3’
NT-3	5’-CATCCACCATCTGTTTGGAA-3’	5’-TGCCGGAAGACTCTCTCAAT-3’
GDNF	5’-AGTAAGGACACCAGCCCTGA-3’	5’-GGCCCAGCTACAGAAAACTG-3’
EGF	5’-TTCCATCTATGTGGGGCTTC-3’	5’-GTAGATGCTGGCCTGTCTCC-3’
CNTF	5’-TTCCAGAAGCGCCATTAACT-3’	5’-GGTGACTTCCATCAGGCAAT-3’
FGF2	5’-TCCGTGACCGGTAAGTATTG-3’	5’-GGCTGCTGGCTTCTAAGTGT-3’
IGF2	5’-AAGCTTTGGATTTTGCCAGA-3’	5’-GTGCCACTGTCCATGTCATC-3’
VEGF	5’-GGAATGGGTTTGTCGTGTTT-3’	5’-GAGAGAGGCCGAAGTCCTTT-3’
TrkB	5’-GCTTATGCCGTGGTGGTGAT-3’	5’-ATGTCTCGCCAACTTGAGCA-3’
ChAT	5‘-CTCGTCCAGAGTATCGGTGG-3’	5’-TGTGAGCCTGAACCTGAAGC-3’
AChE	5’-GACACCGTCTCCAAGGTGAA-3’	5’-CCTGAGGAGAGAGTGTTCCAG-3’
α7nAChR	5’-CACATTCCACACCAACGTCTT-3’	5’-AAAAGGGAACCAGCGTACATC-3’
TNF-α	5’-CACAGAAAGCATGATCCGCG-3’	5’-GAGGCTGAGACATAGGCACC-3’
IL-1β	5’-AGAGCCCATCCTCTGTGACT-3’	5’-GGAGCCTGTAGTGCAGTTGT-3’
ATF4	5’-GATTTCGTGAAGAGCGCCAT-3’	5’-TGCCGGTTTAAGTTGTGTGC-3’
CHOP	5’-GACACCGTCTCCAAGGTGAA-3’	5’-CCTGAGGAGAGAGTGTTCCAG-3’
β-actin	5’-TGCTTCTAGGCGGACTCTTACTG-3’	5’-CTGCGCAAGTTAGGTTTTGTCA-3’
